# India's COVID-19 Burdens, 2020

**DOI:** 10.3389/fpubh.2021.608810

**Published:** 2021-04-14

**Authors:** Ashish Joshi, Apeksha H. Mewani, Srishti Arora, Ashoo Grover

**Affiliations:** ^1^Graduate School of Public Health and Health Policy, City University of New York, New York, NY, United States; ^2^Health and Behavior Studies, Columbia University's Teachers College, New York, NY, United States; ^3^Foundation of Healthcare Technologies Society, New Delhi, India; ^4^Indian Council of Medical Research, New Delhi, India

**Keywords:** COVID-19, India, SMAART RAPID tracker, public health, public policy

## Abstract

The purpose of this article is two pronged; first, to identify and report public health implications of the ongoing coronavirus (COVID-19) pandemic, and second, to report challenges uniquely faced by the citizens of India from a population health perspective. We have done both while closely examining epidemiological data that is accessible via SMAART's RAPID Tracker. This policy informatics platform is a live database aimed to track the geospatial spread of the COVID-19 outbreak and policy actions globally and is administered collaboratively by CUNY's Graduate School of Public Health and Health Policy and a global, non-profit public health incubator. Infectivity, incidence, and recovery rates were computed and graphical representations of epidemiological datasets were studied. We have discussed a plausible conceptual framework based on the principles of population health informatics for countries with similar characteristics to build a stronger public and community health foundation in order to safeguard populations during a health emergency in the future.

## Introduction

**Figure 1:**
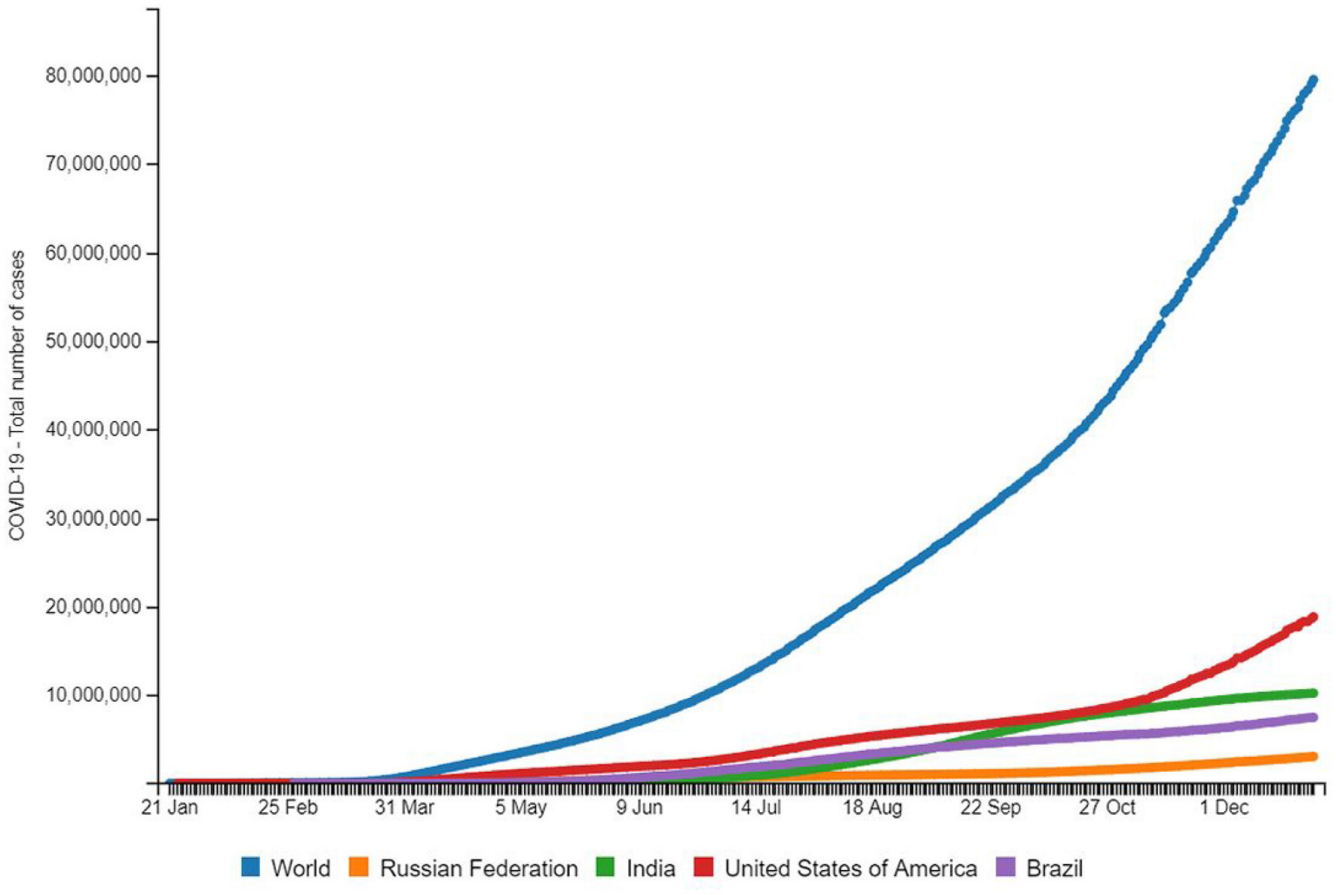
Source: http://www.smaartrapidtracker.org, accessed on December 29, 2020. Total number of cumulative cases of COVID-19 in the world, Russia, United States of America, Brazil, and India (January 21, 2020 to December 29, 2020).

We are in the middle of the first global pandemic of the 21st century and as of December 17, 2020, 72,556,942 COVID-19 cases and 1,637,155 deaths due to COVID-19 were reported worldwide (1, 2). The novel coronavirus that causes COVID-19 was identified in Wuhan, China, in December 2019 (3). By January 30, 2020, the World Health Organization (WHO) declared a global health emergency due to the virus' rapid spread around the world (3). Figure 1 indicates a global spatiotemporal trend of COVID-19 since January 21, 2020 indicating the four countries with the highest caseload—the United States of America, India, Brazil, and the Russian Federation. The dotted lines are observed datasets, and exponential growth trends can be observed for all components of Figure 1, keeping in mind that outbreak statistics behave differently for national and worldwide levels. Looking closely at country-wise incidence, it is indicated that India's total pandemic caseload as of December 17, 2020 was at 9,956,557 making it the second-highest in the world and exceeding that of Brazil as of September 7, 2020. When comparing the spatial and temporal trends of India to the rest of the world in Figure 1, the blazing question arises that even though India is China's immediate over populated neighbor, why was this novel disease late in establishing a foothold in India? We will touch upon this epidemiologic concern in the Discussion section. The data also makes us ponder whether India will surpass the United States of America in recrudescence as it surpassed the Russian Federation on July 6, 2020 with 22,252 new cases as well as with Brazil on September 7, 2020 with 90,802 cases (1). What dynamic roles do India's socio cultural characteristics play in making this a delayed hotspot? How is India prepared to face the burdens of this expanding pandemic? In this paper, we look at underlying factors that determine India's status to tackle a pandemic and also generate informed discussions on some of the mind-tickling

queries that can support and guide public health efforts in the region by analyzing epidemiological data accessed from SMAART (Sustainable, Multisector, Accessible, Affordable, Reimbursable, and Tailored) RAPID (Research-enabled Action-oriented Policy Interventions driven by Data) Tracker.

### SMAART RAPID Tracker

SMAART is a Population Health Informatics (PopHI) framework designed using principles of data, information, and knowledge (DIK); human-centered approach; cognitive fit theory; information processing theory; and humanistic, behavioral, and learning theories (1). As an interactive dashboard, SMAART RAPID Tracker was designed in response to the rapidly changing landscape of both the incidence and mortality of COVID-19 as well as the great variance in policy actions across the globe. The RAPID Tracker is a policy informatics tool using the SMAART informatics framework to track the geospatial spread of the novel coronavirus outbreak and policy actions globally. The platform facilitates the integration of data related to the novel coronavirus with the policy actions of various governments globally. This provides a unique opportunity to evaluate the impact of the policies on the spread of the COVID-19 outbreak.

As an interactive dashboard, SMAART RAPID (Research enabled Action oriented Policy Interventions driven by Data) Tracker designed in response to the rapidly changing landscape of both the incidence and mortality of the novel coronavirus (COVID-19) as well as the great variance in policy actions across the globe. The platform facilitates integration of the novel coronavirus (COVID-19) related data with policy actions of various governments globally. The dashboard aggregates publicly available but verified information on the burden of the novel coronavirus (COVID-19) as well as aggregating policies/advisories and the timeline in which they were enacted for each country. This structure emphasizes the importance of considering both the epidemiological and political realities to understand what types of non-pharmaceutical interventions are effective. This becomes even more important with the resurgence of cases in countries and areas that previously experienced declines.

SMAART Rapid Tracker has 4 modules including (a) a data module that gathers COVID-19 related data on community wide transmission, total and new confirmed cases, recovered cases, and total and new fatality rates across global settings; (b) a policy module that facilitate users to examine the impact of policies and advisories on COVID-19 trends; (c) an insights module that aims to track trends and analysis of COVID-19 globally; and (d) a digital resource module that aims to aggregate national and global digital resources available that are related to COVID-19. SMAART Rapid Tracker is operational on Wordpress, an open source platform. The technology stack includes HTML, CSS, and JavaScript libraries as frontend, PHP 7.3 as server-side script, and MySQL 8.0 as the database. The dashboard is cross browser compatible and completely responsive on mobiles and tablets. The SMAART RAPID Tracker id designed using principles of an existing human-centered, geovisualization platform, SanaViz, an Internet-enabled, interactive app incorporating principles of human-centered design and cognitive fit theory to enhance visual exploration of population health data. In addition, similar principles have been used to design and develop the ETE dashboard tool developed to track New York's progress toward achieving the goal of its ETE initiative, to reduce new HIV infections from 3,000 per year to 750 per year by the end of 2020.

The immense burdens placed on the most vulnerable groups entangle this image undeniably, as brought to light by relevant epidemiologic trends. Developing proof demonstrates disability and incapacity due to the coronavirus for racial-ethnic minorities at disproportionate levels given the comparative transmission rates among other segments of population around the world (4). The presymptomatic and asymptomatic spread has caught us by surprise and has led to a rapid spread of infection while scientists are still trying to explore the constantly mutating novel virus.

### Epidemiology of COVID-19

In 2020, Bill Gates indicated in a TED interview that the transmission dynamics of COVID-19 are known to be more difficult than what experts predicted (5). The first cases were reported in December 2019, and from December 18, 2019 to December 29, 2019, five patients were hospitalized with acute respiratory distress syndrome, and one of these patients died (6). India reported its first COVID-19 case on January 29, 2020 from a group of students returning from Wuhan, China, to Kerala (7). Within 5 days, the number recorded increased to three and stayed the same until March 2020 (6, 7). Throughout February, no major control measures were taken other than temperature screening of people returning from China at major airports. On March 4, 2020, the number of cases increased to 22, including 14 Italian tourists (7).

An understanding of the virus' genomic attributes helps discern its pathogenesis. “Genome Composition and Divergence of the Novel Coronavirus (2019-nCoV)” shows significant differences between SARS or SARS-like CoV and COVID-19 (8). COVID-19 is mainly transmitted through respiratory droplets or fomites from an infected person via mucous membranes of the mouth, nose, and eyes (9), with the average incubation period ranging from 5 to 6 days (9). The risk of transmissibility of COVID-19 to the reproduction of the virus (Ro) is 3.28, much more than the WHO's range of 1.4–2.5 (10). Ro is an indication of the transmissibility of a virus representing the average number of new infections generated by an infectious person in a vulnerable population (for R0 > 1, the number of infected individuals is likely to increase, and for R0 < 1, the transmission is likely to die out). The basic reproduction number is a central concept in infectious disease epidemiology indicating the risk of an infectious agent concerning epidemic spread (9); it indicates that around three persons will be infected by an index patient. These reproduction estimates of the infectious agent in a population of 1,380,937,553 (making it 17.7% of the world population) estimated as of July 27, 2020 based on Worldometer elaboration of the latest United Nations (UN) data, with a population density of 1,202 people per square mile (10), suggests high priority, timely national responses from the government. Was the government of India prepared for this?

## Challenges Unique to India

### Government Action—Boon or Bane?

In a country of ~1.3 billion people, Hinduism is the most common religion in India, accounting for about 80% of the population; Islam is the second-largest religion comprising 13% of the population; and other major religious groups in India include Christians (2.3%), Sikhs (1.9%), Buddhists (0.8%), and Jains (0.4%) (11). Due to the socio-economic pattern and cultural and religious values of India, the challenges posed by COVID-19 are very different compared to its European counterparts. On March 19, 2020, at a point prevalence of 500 (1), Prime Minister Narendra Modi announced a “janata curfew” for March 22, 2020, in what was seen as a mock drill to prepare citizens for a longer lockdown in the future (12, 13). This lockdown was aimed at stalling the spread of the virus. The lockdown in India represents a massive logistical and implementation challenge given the population size and its density. Despite the government's measures to address crises like distributing food on a large scale, pressing employers to pay wages, and landlords to waive rents, panic and uncertainty especially among the migrant laborers resulted in them traveling, sometimes even by foot, hundreds of miles to their villages back home.

India's largest lockdown ever began with a 4-h notice, when Narendra Modi announced at 8 p.m. on March 24, 2020 that the entire country would be brought under curfew from midnight to curb the spread of COVID-19 (13). At the time, there were only 320 recorded cases confined to a few regions and 10 deaths in a population of more than 1.3 billion (1). In a matter of months, India became one of the worst-affected countries globally (14). This increase has been attributed to augmented testing and spread of the infection, despite one of the most stringent lockdowns in the world.

Information collected from media sources yielded a government response that included a prolonged lockdown, a public awareness campaign, and a series of innovations including a novel smartphone application called Aarogya Setu for the purpose of contact tracing and aiding in quarantine and related containment measures (15). After assessing the government's actions closely, the question “if India had handled the pandemic differently, would it be in a better position?” arises. In this review we look at the COVID-19 situations in Vietnam and Pakistan and also propose a tool in the form of an asset-based framework that would assist decision makers as there seem to be complex multidimensional forces at play.

### Geopolitical Challenges and Socio-Economic Impacts

In December 2020, India witnessed the largest protest of this century; an estimated 250 million farmers traveled hundreds of miles in solidarity with strikers, protesting against three new agricultural laws that were drawn in September (16). These laws were enacted under the auspices of modernizing the agricultural industry and permitting entry of big agro-business corporations in agriculture and therefore weakening the already shaky structure of the Indian democracy (16). Approximately 65% of the India population is associated with agricultural professions (17). Suicide rate for farmers are higher than other sectors globally (17) and the current protests by unmasked farmers during a pandemic is expected to have adverse outcomes given the etiology of COVID-19. A total of 10,224,303 COVID-19 cases have been reported in India as of December 29, 2020, of which 761,494 are new cases in the month of December 2020 alone (1).

**Figure 2:**
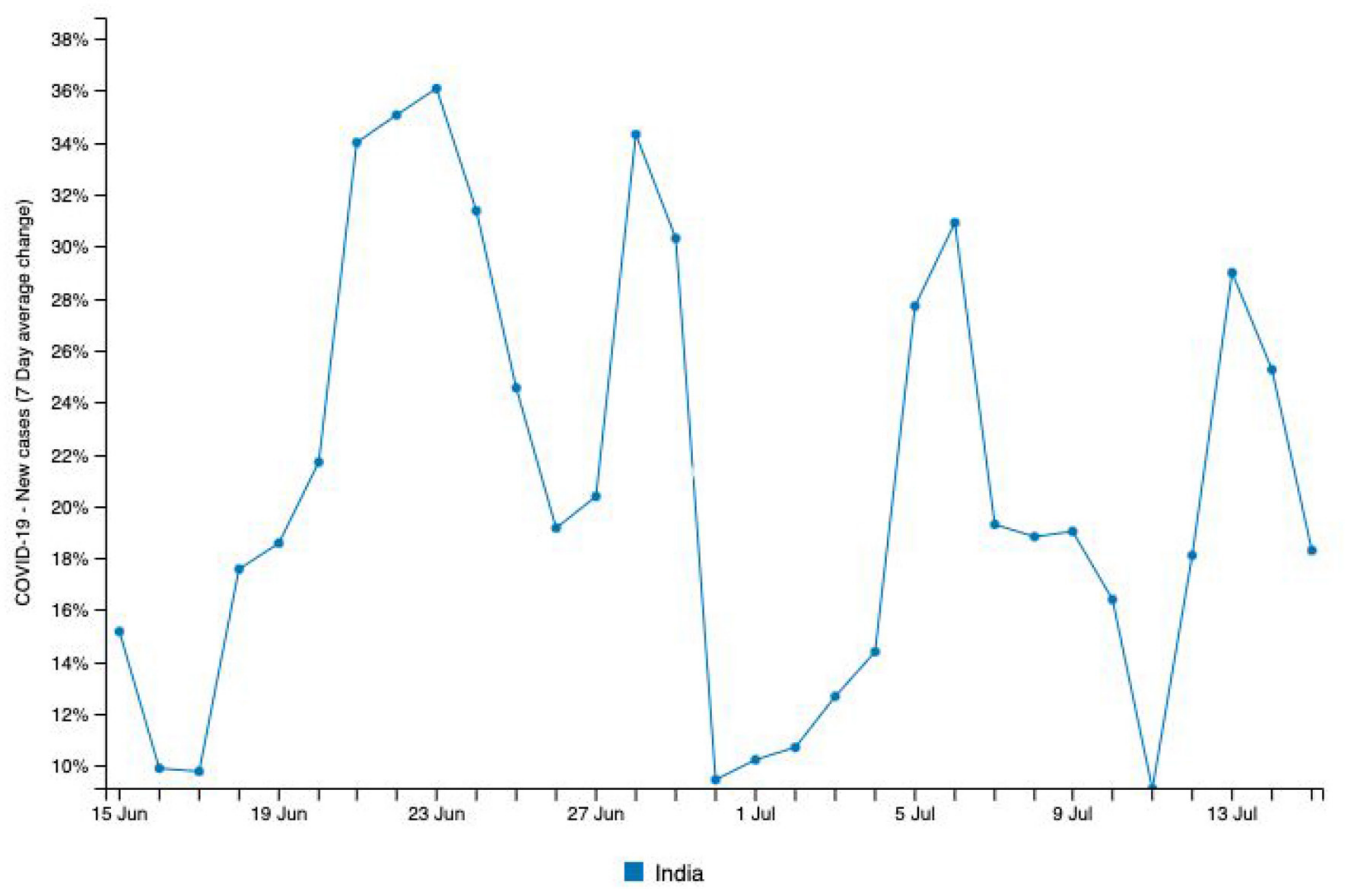
Source: http://www.smaartrapidtracker.org, accessed on July 27, 2020. The New cases (7 Day average change) of COVID-19 in India (June15, 2020, to July 15th, 2020).

The contentious Indo-Chinese situations during this pandemic are considered to be one of the worst geopolitical challenges currently ongoing in Asia. On June 15, 2020, China initiated an attack on armed forces at the Indian border amidst an ongoing pandemic (18). It triggered a series of protests that brought people into the streets to campaign against the use of Chinese products and software applications. Mass gatherings affected the observation of social distancing guidelines from the local, state, and central health authorities, which subsequently impacted overloaded medical systems and healthcare personnel. Figure 2 shows a spike of new cases (36.08%) recorded around June 24, 2020 in a 7-day average change in new cases from June 15, 2020 to July 15, 2020 (1). Considering an average incubation period of 5–6 days, data suggests that these mass gatherings could have been one of the factors that triggered higher infection rates.

In addition to India's healthcare system being adversely affected by the current pandemic, international relations have also suffered a setback. According to the Ministry of Statistics and Program Implementation in India, the growth rate of the country has dropped to 3.1% (14), and the UN report “The World Economic Situation and Prospects” as of mid-2020, projects India's growth rate to fall to 1.2% in 2020 (19). The trade impact for India is estimated to be ~348 million dollars and the figures indicated India to be among the top 15 economies most affected as slowdown of manufacturing in China disrupts world trade (19). For India, the trade impact is estimated to be the most for the chemicals sector at 129 million dollars, textiles and apparel at 64 million dollars, automotive sector at 34 million dollars, electrical machinery at 12 million dollars, leather products at 13 million dollars, metals and metal products at 27 million dollars, and wood products and furniture at 15 million dollars (19, 20). This has come as a direct consequence of the spread of coronavirus (COVID-19). When we see China's share in total imports to India, India's total electronic imports account for 45% of China. Around one-third of machinery and almost two-fifths of organic chemicals that India purchases from the world come from China. For automotive parts and fertilizers China's share in India's import is more than 25%. Around 65–70% of active pharmaceutical ingredients and around 90% of certain mobile phones come from China to India.

India's Chief Economic Advisor Dr. Krishnamurthy Subramanian said that trade between India and China—which grew from $3 billion in 2000 to an all-time high of $95 billion in 2018—is largely in China's favor (20). China's exports to India are four times higher than its imports from India. India's trade deficit with China is the single largest it runs with any country as Chinese investors poured millions of dollars into India's largest new-age companies (20). These economic and internal security changes provide compounded roadblocks that are unique to India in dealing with this pandemic.

Given the nature of the disease which is highly contagious, the ways to contain the spread include policy actions such as imposition of social distancing, self-isolation at home, closure of institutions, and public facilities, restrictions on mobility, and even lockdown of an entire country. These actions can potentially lead to dire consequences for economies around the world. In other words, effective containment of the disease requires the economy of a country to stop its normal functioning. A stimulus of 20 trillion Indian rupees was passed as a response to this health crisis (21). The country's corporate credit in June 2019 was greater than that of June 2020, suggesting that banks did not access much of this emergency fund. Recuperation through investment has stalled and companies have deleveraged resigning old obligations (21). The use of electricity, petrol, and diesel are beginning to recover post the initial lockdown lows but have still been 10–18% below that of what it was in June 2019 (22). There has been a 23.5% spike in unemployement that continues to asscend margianally (22). Due to the lack of infrastructure, although over half of India has access to smartphones, relatively few can work remotely. Jobs related to retail and manufacturing require physical presence and interaction and have been directly impacted (21, 22). Although the Mahatma Gandhi National Rural Employment Guarantee Act (MNREGA) supports 100 days guaranteed employment, it does not cover urban areas. Critically, the larger firms are perceived healthier. Laborers are struggling to get paid by the micro, small, and medium-sized enterprises (MSMEs) that are intermediate inputs and service suppliers to the modern sector (21, 22). However, small and micro enterprises, which are the largest source of employment outside agriculture, have minimal access to formal credit and constitute 99.2% of all MSMEs (22). Their inability to bounce back could see India face further economic and social tensions. The economy is withstanding both supply and demand shocks with the wholesale prices index declining sharply (21, 22).

### Population Density and Weather

India's high population density brings together another challenge in managing this widespread pandemic. It's two megacities, New Delhi and Mumbai have a population density of 29,259.12 and 73,000 per square mile, respectively, being some of the most densely populated cities in the world (11). Because property prices are at such a premium, residents of Mumbai frequently live in low-cost, cramped housing located far from their workplace, leading to long commutes on the city's busy mass transit system. Comparatively, New Delhi covers a larger area than Mumbai (11). Social distancing in such densely populated cities along with 10–15% of these cities' populations being illiterate coupled with cultural practices that facilitate gathering in groups might contribute to the emerging infectivity rate (23). For these dense communities in India, inadequate shelter and overcrowding are also some of the high risk factors aiding in transmission of the virus. According to a recent report by the National Centers for Disease Control (NCDC), unauthorized colonies and jhuggi-jhopri clusters pose a serious problem as a large number of people live in these colonies (24). A “jhuggi-jhopri” refers to a small roughly built house or shelter usually made of mud, wood, or metal that has thatched or tin-sheet roofs and a “slum” refers to an area consisting of poorly built, overcrowded clusters (17). Residents of these inadequate housing facilities usually lack access to adequate sanitation facilities, and self-isolation is often impossible.

While attempting to fight off the coronavirus, India has also been suffering a heat wave that has worsened the crisis as residents struggle to stay home. Temperatures soared to nearly 110° Fahrenheit in New Delhi in late May 2020, making wearing a mask unbearable to some and social distancing harder to maintain in close proximal housing settings (10). This heatwave has definitely dealt a setback while dealing with the virus; additionally, India had to also deal with devastating floods and landslides triggered by torrential rains in various parts of the country. The hard-hitting monsoon in India has affected 16 states and millions of people, resulting in a loss of roughly 900 lives while disrupting normalcy (25). The highest number of deaths this monsoon due to floods and landslides was recorded in the state of West Bengal where 239 people died, followed by 136 in Assam, 87 in Gujarat, and 74 each in Karnataka and Madhya Pradesh (26). On August 7, 2020, heavy rains majorly impacted the southern state of Kerala and claimed the lives of 49 people (26). Approximately six million people in Bihar and over five million people in Assam have been displaced and have abandoned their livestock and livelihood (26). Rivers are flowing above the danger level in almost all states making it difficult to monitor the exact number of casualties in those areas and aggravating the suffering of families of missing persons. Displaced persons taking shelter in refugee camps deployed by the National Disaster Response Force (NDRF) and the State Disaster Response Forces (SDRF) are making social distancing guidelines recommended by the center very difficult to follow (26, 27). Refugee camps make physical isolation impossible and people live in insanitary and inhospitable conditions. Sometimes, up to six families live in one tent within a 3 m^2^ area (27). Limited infrastructure to deal with COVID-19 in these camps puts these vulnerable populations at an even greater risk. According to Nott (2020), “apart from difficult living conditions in these camps, many people share one latrine and washing facilities and hundreds queue for food every day” (27). The deluges in India cause similar damage every year, pushing millions into greater poverty due the loss of habitat, creating a snowball effect. This condition is proving dire for the disease burden.

### Testing and Recovery

**Figure 3:**
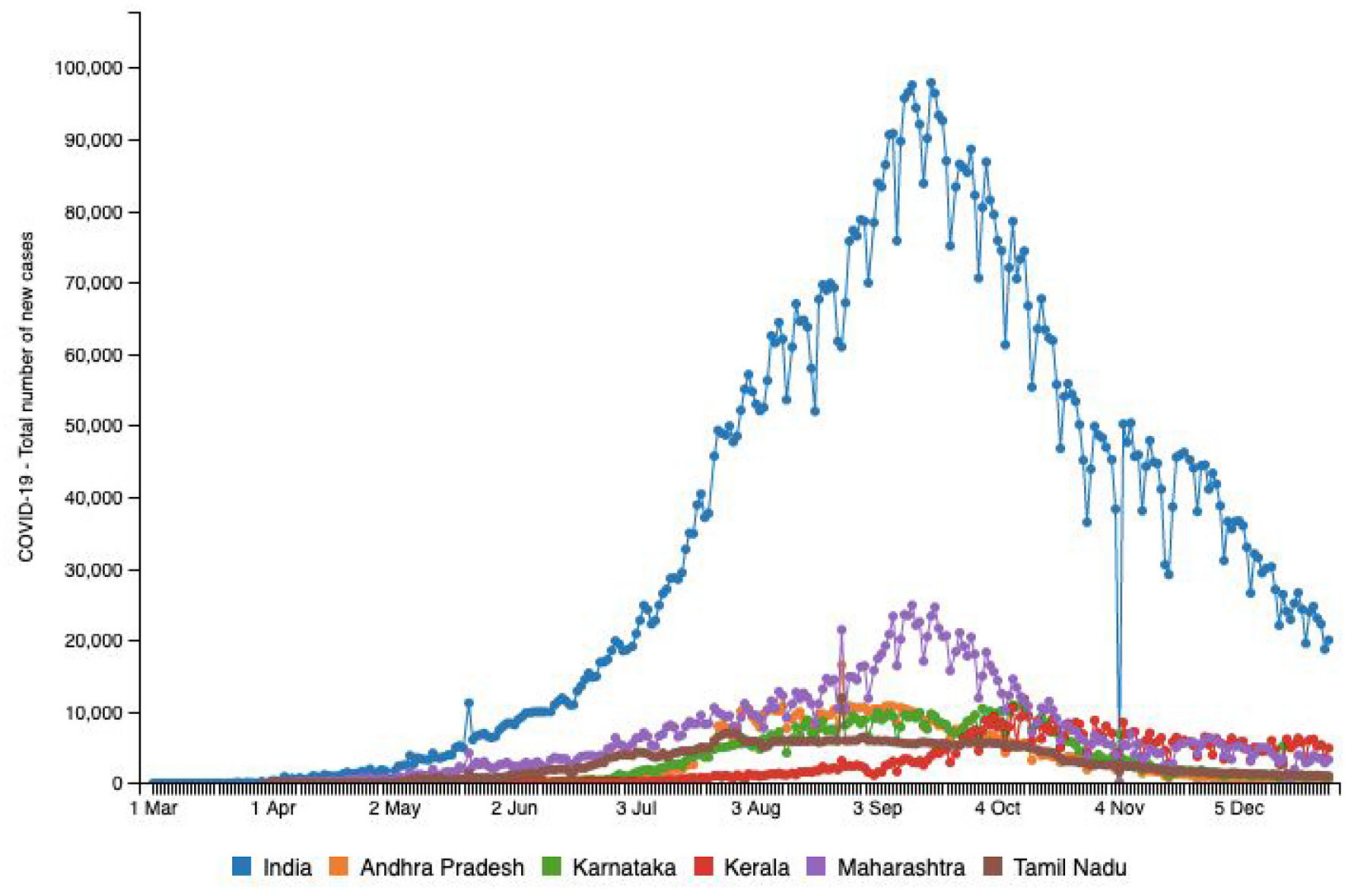
Source: http://www.smaartrapidtracker.org, accessed on December 29, 2020. Status of COVID-19-Total number of new cases in India, and in the states of Maharashtra, Andhra Pradesh, Delhi, Karnataka, Uttar Pradesh, and Tamil Nadu (March 1, 2020 to December 29, 2020).

According to data gathered from SMAART RAPID Tracker, the incidence rate of COVID-19 recorded in India is calculated at ~35.73% (per million) (1). The infectivity rate of COVID-19 in India has been calculated at around 0.9% with ~817,209 recovered cases and a total fatality of 30,601 cases as of August 6, 2020 (Figure 3) (1). These inferences are based on data available from laboratory testing and diagnosis for identification of COVID-19 cases, which is critical in order to identify and isolate positive patients to contain the spread of the pandemic. Most treatment decisions in clinical settings are based on laboratory outcomes. In India, the availability of tests was identified as a challenge when the pandemic just hit the country; an average of only 1,500 samples were tested daily until early April 2020 (28). While health experts noted that efficient testing would be vital to containing the spread of COVID-19, inadequate testing data from India before mid-April suggests delayed identification and isolation of positive cases. Literature reports that contact tracing of identified cases and quarantining of those infected can slow the spread of infection. Diagnostic testing for COVID-19 which helped companies supply their own tests to government and private laboratories, hospitals, and other clinical settings served as cushioning in safeguarding growing testing capabilities.

A total of 1,415 operational laboratories in India are testing for COVID-19 as of August 10, 2020; Real-time polymerase chain reaction (RT-PCR): 720 (government: 431 + private: 289), TrueNat Test: 584 (government: 481 + private: 103), and CBNAAT Test: 111 (government: 32 + private: 79; 21) (28). The states of Maharashtra, Tamil Nadu, and Andhra Pradesh have reported the highest number of cases as of August 10, 2020 (1). **Table 2** provides a snapshot of the testing capabilities in the few highly impacted states of the country. This table helps us understand the availability of Indian laboratories in testing samples to identify and isolate positive cases. Maharashtra is known to be the hardest hit with a burden of 1,928,603 cases as December 30, 2020 (1). This western state is considered a hotspot that accounts for nearly one-third of the total cases as well as ~40% of deaths in India (1). Even though the fatality rate of 4.3% is lower than that of the rest of the world, it is significantly higher than that of the other Indian states (29). As of August 10, 2020, there were a total of 142 pathology laboratories for coronavirus testing in Maharashtra, including 77 government and 65 private laboratories (28). Mumbai, the largest city in the state of Maharashtra, is the most densely populated city in the entire country and has constantly had the highest number of COVID-19 cases in the country.

**Figure 4:**
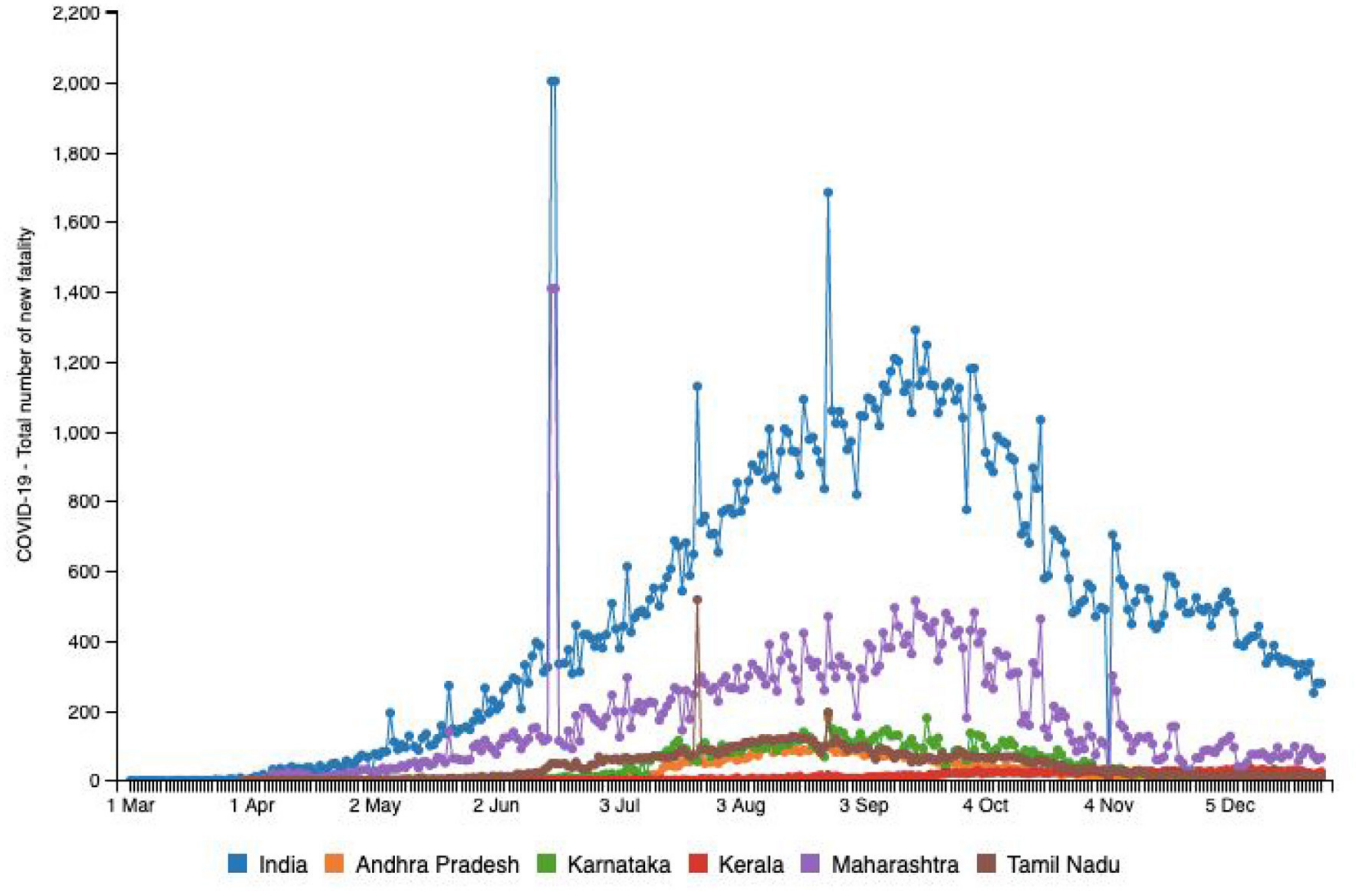
Source: http://www.smaartrapidtracker.org, accessed on December 29, 2020. Status of COVID-19 Total number of new fatality in India, and in the states of Maharashtra, Andhra Pradesh, Delhi, Karnataka, Uttar Pradesh, and Tamil Nadu (March 1, 2020 to December 29, 2020).

Both Tables 1, 2 help us identify a possibility of correlation between testing availability and incidence and mortality caused by the virus. Areas that are robustly testing are able to identify and curb the growing trends, and hence, testing availability at the right time is crucial. A multitude of factors such as testing availability, laboratory facilities, and caveats in testing and data reporting contribute to the burdens of India's pandemic support structure. In the early stages of the pandemic, testing kits were not easily available and were used only in symptomatic cases following strict guidelines and in select public testing facilities. A BBC report indicated that the most common test used in India is the RT-PCR test that isolates genetic material from a swab sample (30). While they are considered the benchmark of COVID-19 testing, they are India's most expensive test, taking 8 h to process and up to 24 h to provide results, depending on transport time to laboratories (30). In a bid to boost testing capacity, India opted for rapid antigen tests, commonly known as diagnostic or rapid tests. These are cheaper, and by isolating proteins (called antigens) that are unique to the virus can offer results in just 15–20 min (30). However, the Indian Council of Medical Research (ICMR) evaluations found that their accuracy rates were as low as 50%, while the All-India Institute of Medical Sciences (AIIMS) found their accuracy in giving a true negative result ranges from 50 to 84% (28). Figure 3 highlights the number of new cases reported in the above-mentioned six states in comparison with country-wide data until December 29, 2020. Figure 4 provides an outline of new fatalities reported in the same states until December 29, 2020. Comparing the slopes of Figures 3, 4, we can propose a compelling rationale for strong consideration of proportionality in new cases against new fatalities (1). Infections are rapidly rising in these states, yet the recovery rate continues to rise and now stands at ~63.5% (1). Can we credit India's ancient Ayurveda and alternative medicine practices to this growing recovery rate or the median age of 28.4 years making it one of the youngest populations in the world enabling uncompromised immune systems?

**Table 1:** Source of Data: http://www.smaartrapidtracker.org, accessed on August 6, 2020.

**Country**	**Population**	**Total tests**	**Total cases**	**Total recovered**
USA	331,192,837	62,768,388	5,000,443	2,552,190
Brazil	212,706,570	13,329,028	2,873,304	2,020,637
India	1,381,307,956	22,149,351	2,025,409	1,377,384
Russia	145,940,753	29,716,907	871,894	676,357
South Africa	59,381,566	3,149,807	529,877	387,316
Iran	84,097,623	2,612,763	320,117	277,463
UK	67,922,029	17,515,234	308,134	N/A
Italy	60,452,568	7,099,713	249,204	201,323
Germany	83,811,260	8,586,648	215,100	196,200
China	1,439,323,776	90,410,000	84,565	79,088

*Table with testing numbers, new cases, and recovered cases for some highly affected countries as of August 6, 2020*.

**Table 2:** Source of Data: Indian Council of Medical Research https://www.icmr.gov.in/, accessed on August 10, 2020.

**State**	**Govt. labs**	**Private labs**	**Cases per million**	**New cases**	**Total recovered**	**Total fatality**
Maharashtra	77	65	4,586	12,248	351,710	17,757
Tamil Nadu	61	70	4,116	5,994	238,638	4,927
Andhra Pradesh	67	9	2,691	10,820	138,712	2,036
Karnataka	45	56	2,913	5,985	93,908	3,198
Delhi	23	39	8,681	1,300	130,587	4,111
Uttar Pradesh	120	40	614	4,571	72,650	2,069

*Table with laboratory numbers, cases per million, total recovered, and total fatality for some highly affected Indian states as of August 10, 2020*.

### COVID-19 in Vietnam and Pakistan

**Figure 5:**
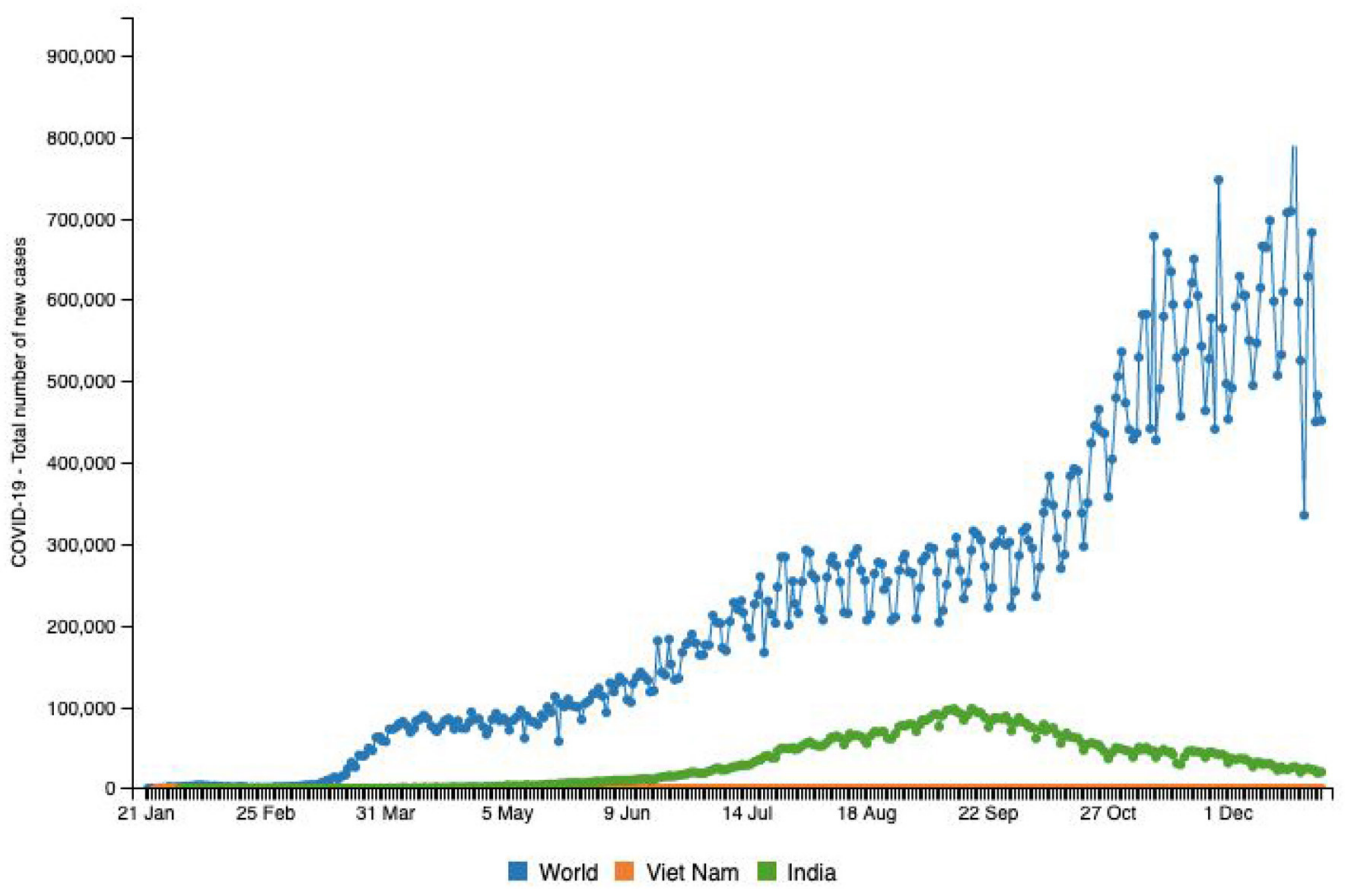
Source: http://www.smaartrapidtracker.org, accessed on December 29, 2020. Total number of new cases of COVID-19 in the world, Vietnam, and India (January 21, 2020 to December 29, 2020).

**Figure 6:**
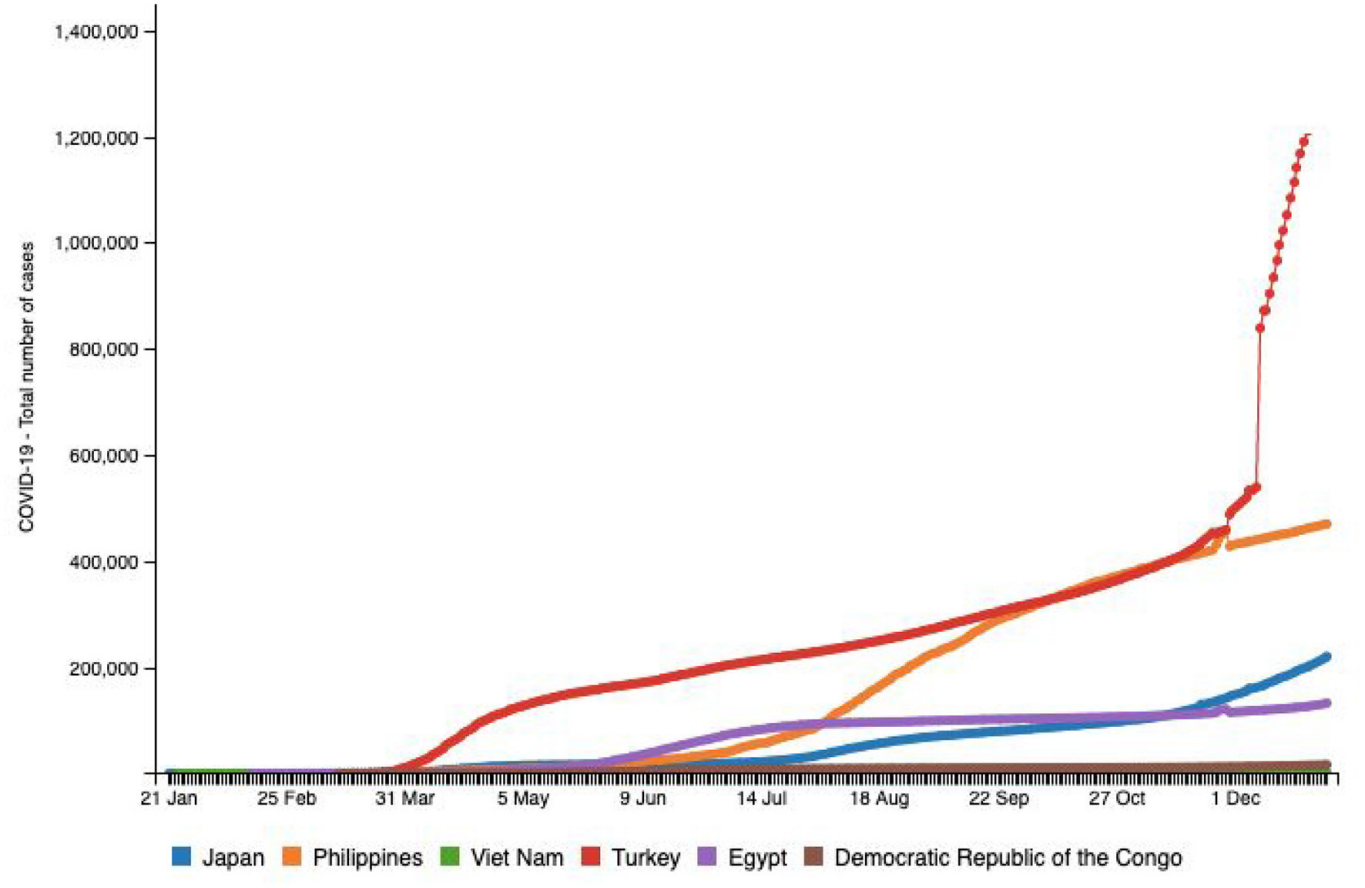
Source: http://www.smaartrapidtracker.org, accessed on December 29, 2020. Total number of cumulative cases of COVID-19 in the world, Vietnam, Japan, Philippines, Turkey Egypt, and Congo (January 21, 2020 to December 29, 2020).

China borders an expansive 795 miles of Vietnam and the shortest distance via air between the two countries is 1,313.41 miles (10). Vietnam was at a high risk owing to its proximity to China and the high rate of coronavirus transmission. However, two cases were reported on January 24, 2020 (1), and only 1,414 cumulative cases by December 22, 2020 (1). Figure 5 helps us visualize the relatively low incidence rates in this middle-income, developing Asian nation, raising curiosity about the government's policies and procedures to tackle the spread of COVID-19, given that it is a nation with limited resources and a high population (1). While the rest of the world is still grappling with the severity of rising infections, Vietnam has demonstrated a strikingly low number of fatalities of only 35 people until December 22, 2020 in comparison with Pakistan and India at 9,392 and 146,111, respectively (1). As seen in Figure 6, the rate of infection in Vietnam has been evidently much lower in comparison with nations like Japan, Turkey, the Philippines, Egypt, and Congo, that have similar populations (10). Vietnam's policy responses to the outbreak was reviewed by La et al. (2020), and they identified 173 official instructions, guidelines, plans, dispatch, policies, and direct actions that were issued by the government until April 4, 2020 (31). A couple of days after China affirmed the flare-up of COVID-19, the Ministry of Health (MOH) in Vietnam issued a mandate for identified cases to isolate (32). On January 10, 2020, the Public Health Emergency Operation Center held a briefing to assess the situation and respond (31, 32). The website (http://ncov.moh.gov.vn) was launched as an innovative initiative. The NCOVI and Vietnam Health applications were introduced to supply nationals with timely data and live-chat features (31). On February 1, 2020, public authorities announced an emergency because six new cases had been identified. What followed were severe measures to keep the infection from spreading, including isolation, disconnection of suspected infection transporters, and willful seclusion at the network (32).

**Figure 7:**
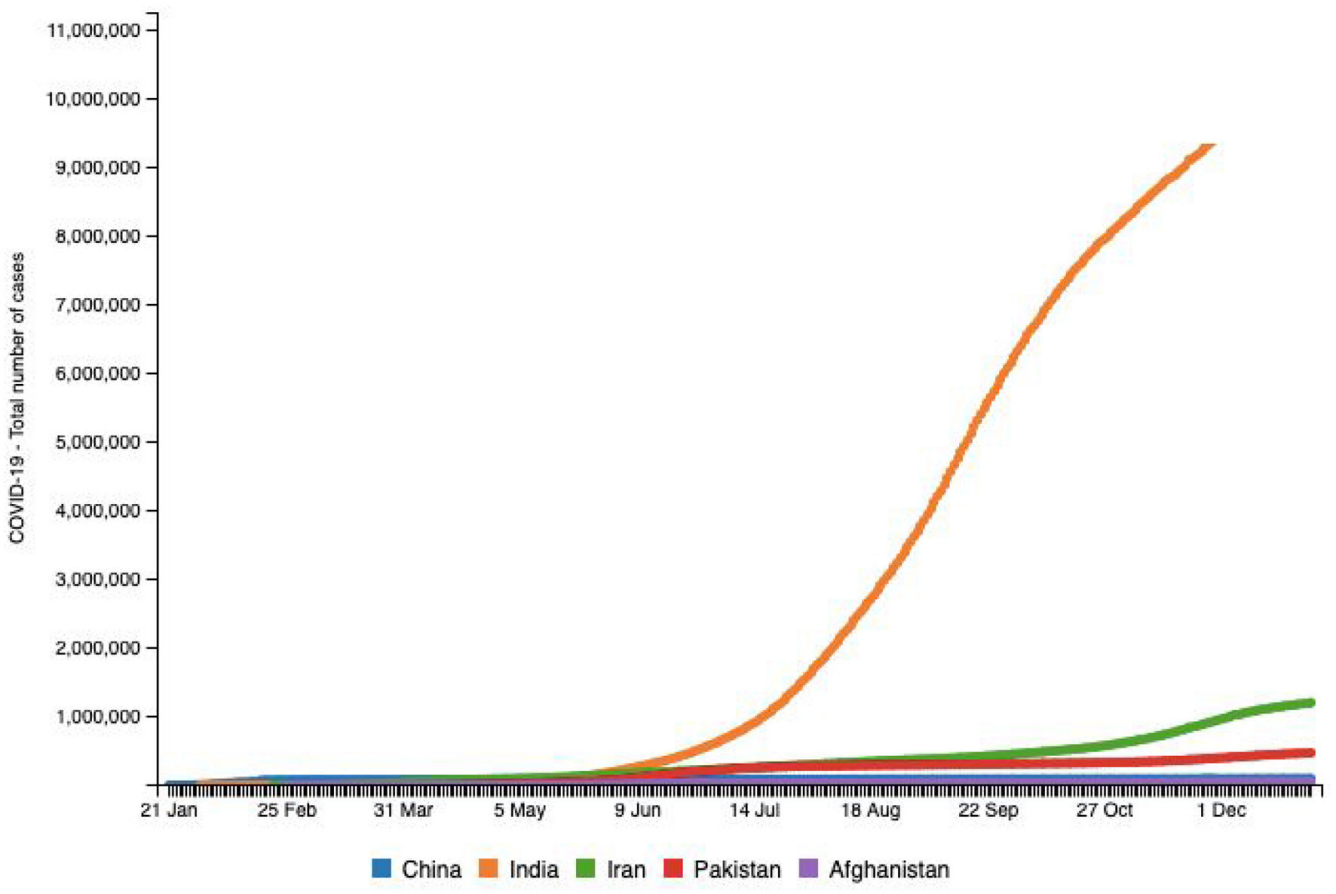
Source: http://www.smaartrapidtracker.org, accessed on December 29, 2020. Total number of cumulative cases of COVID-19 in the world, China, India, Iran, Pakistan, and Afghanistan (January 21, 2020 to December 29, 2020).

Pakistan, with ~212 million inhabitants, is the fifth most populous country in the world and shares its borders with China, India, Afghanistan, and Iran (11). The first COVID-19 case was confirmed by the Ministry of Health, Government of Pakistan, on February 26, 2020 in Karachi, Sindh province (33). By December 22, 2020, Pakistan had confirmed 458,968 cumulative cases (Figure 7). The Government of Pakistan has established a COVID-19 Relief Fund to receive donations for public welfare. Social network helplines were launched by the government in seven local languages (33). Every medical organization that was supporting the treatment of COVID-19 positive patients was required to conduct “need and availability assessment” of supplies equipment, personal protective equipment (PPE), and laboratory diagnostics, including identification of sources to ensure provision and availability of these (34). Pakistan also observed an uptick in suicidal rates during and after the pandemic, most of these suicides occurring due to a lockdown-related socioeconomic distress and the fear of infection (34, 35). MSMEs were also severely victimized (35). In a study conducted by Shafi et al. (36), two-thirds of participating enterprises reported that survival would be difficult if the lockdown lasts more than 2 months (36). There was also a disruption in routine immunization and other health services, which adversely impacted the common people (37). Another worry is the impact of sewage waste and drainage on groundwater as the presence of COVID-19 in stool has been fundamentally revealed in literature (38). Groundwater pollution is getting more serious in nations like India, Bangladesh, and Pakistan, where waste is released into water bodies (38). A host of impurities and microorganisms weaken groundwater quality, and the presence of stool in sewage channel water prompting groundwater contamination can be an arising danger and could facilitate further spread of COVID-19 (38). This plague has also caused the disruption of other health care services, routine immunization being one of them. This could possibly onset secondary outbreaks of vaccine-preventable diseases and eventually exacerbate immunization disparities (39).

## Need for a Population Health Informatics Surveillance platform

A conceptual framework is an analytical tool with the ability to be applied to various settings. As seen in the literature, the COVID-19 pandemic suffering has been heightened due to human rights failures, but the right to health can provide a base for guaranteeing that the pandemic response serves to realize the right to the highest attainable standard of physical and mental health for all (40). The CDC provided a framework to local governments to respond to the plague (41). The framework aims to protect high risk individuals (e.g., 65 years of age and above, those with underlying medical or health conditions, etc.), vulnerable populations (e.g., refugees, internally displaced persons, prisoners), first responders, healthcare personnel, and critical infrastructure workers (41). Adapting interventions such as these, if supported by existing public health programs helps address the immediate mitigation needs. The UN provides a methodology and outline to dire financial reactions of the pandemic, following the Secretary-General's report on the socioeconomic impacts of the COVID-19 emergency (42). This tool's emphasis is placed on the present time and setting at a national level. The UN development system (UNDS) is supporting wellbeing frameworks, food security frameworks, reestablishing and working to better their fundamental and social administrations, and adopting unique measures to limit the effect of the pandemic on the most weak populaces (42). The UNDS themselves is ensuring the implementation of framework during the COVID-19 emergency; simultaneously, it is helping secure individuals through social insurance and ensuring occupations, little, and medium-sized undertakings, and the weak specialists in the casual area through monetary recuperation. The framework is also helping direct an important flood in financial and monetary boost to make the macroeconomic structure work for the most defenseless, encourage supportable turn of events, and reinforce multilateral and territorial reactions (42). This will fabricate trust through social discourse and political commitment as well as put resources into a network driven flexibility.

For surveillance of COVID-19 and its cause, the implementation of syndromic surveillance and commercial laboratory reporting needs to be designed and executed to address the gaps. A population health informatics surveillance system has the ability to draw from a combination of data sources and create an updated, more accurate picture of the disease's spread, its effects, and generate specifics necessary to inform the national public health response to COVID-19. Based on our understanding of the challenges faced by developing Asian nations, it is critical to have robust surveillance in place to control the spread of COVID-19 as a system of this nature will enable rapid detection, isolation, testing, management of suspected cases, application of prevention measures, and detection and containment of further outbreaks among vulnerable populations. It will also support evaluating the impact of the pandemic on the health-care systems and society, monitoring long term epidemiologic trends and the evolution of novel diseases, and assess the association between COVID-19 and other viruses (43).

**Figure 8:**
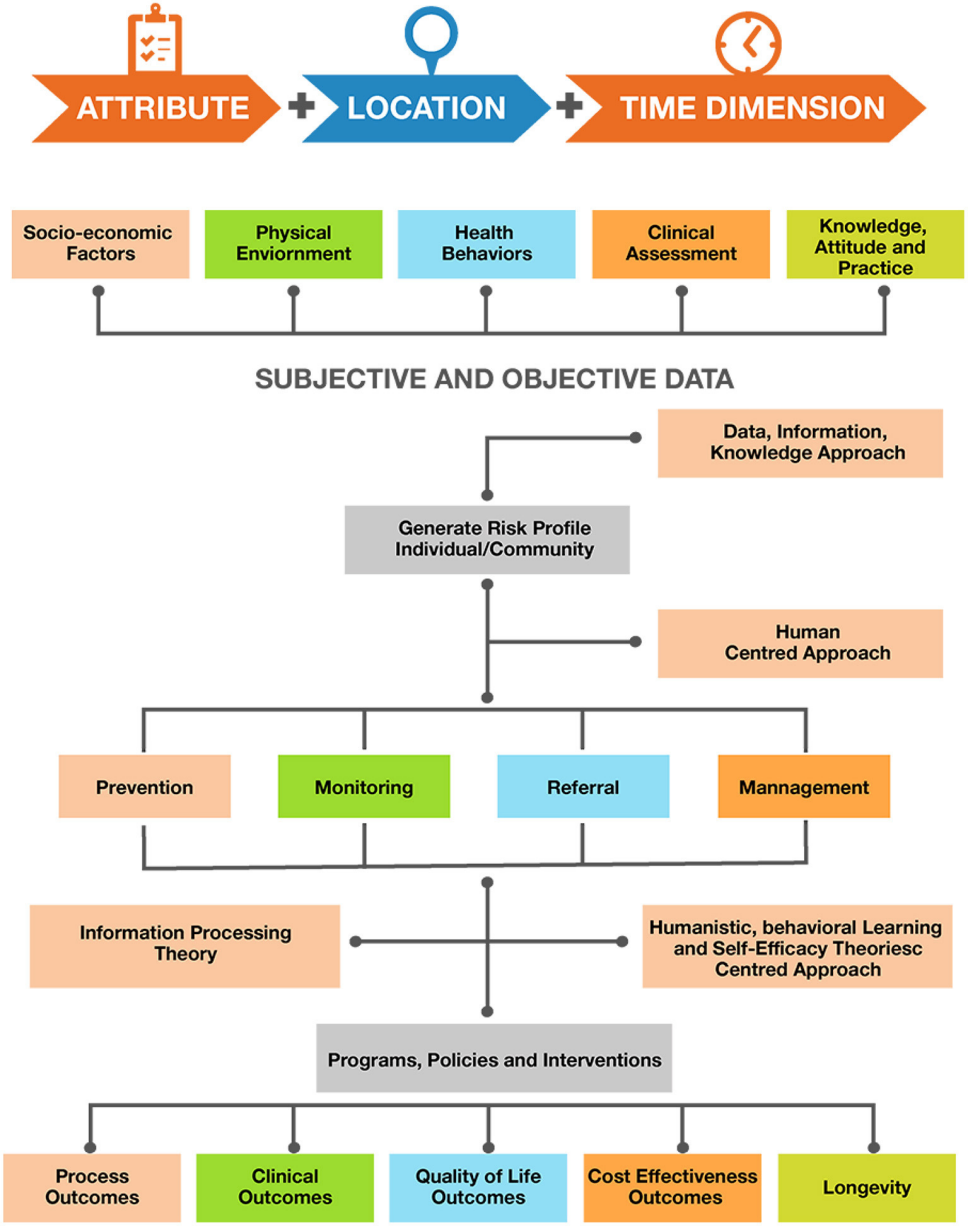
Population Health Informatics Surveillance System.

**Figure 9:**
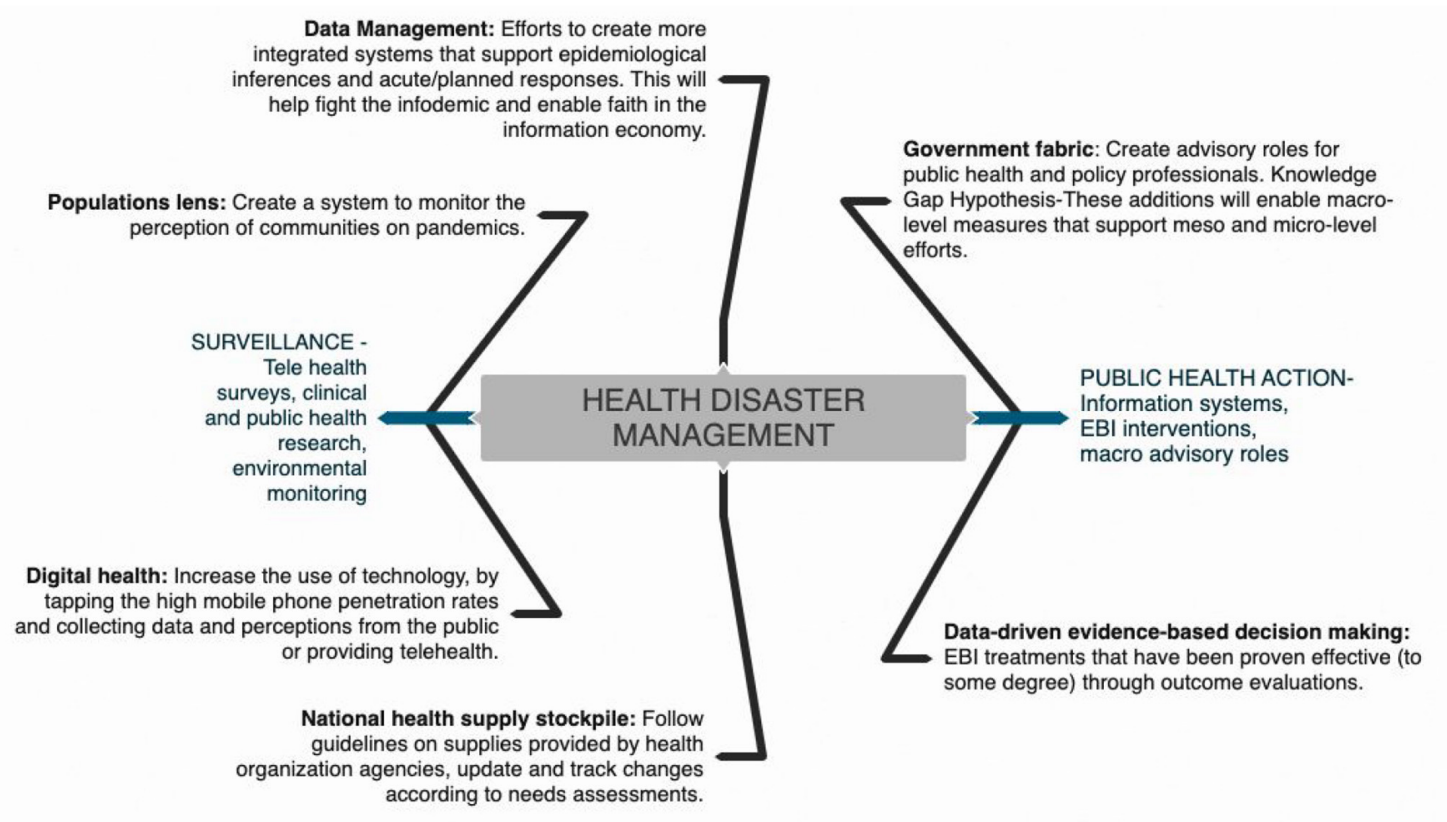
Framework to support decision making for future pandemic preparedness.

On the policy level we suggest that apart from the spatiotemporal data collection and case notification of COVID-19 as proposed by WHO, information regarding signs and symptoms of COVID-19 and laboratory assessment combined with information related to individual socio-demographics, physical environment, health behaviors, additional clinical assessments, and knowledge attitude and practices should also be recorded. As discussed, there are a multitude of factors that influence the outcome of the COVID-19 pandemic. Information on socio-demographic variables (like age, gender, education, and income) and physical environments (like built in environments such urban, rural, or slum settings) is also essential to record and determine both at risk populations and vulnerable settings. Data on health behaviors including variables such as sleep patterns, alcohol consumption, diet, and physical activity patterns are essential to determine their association with individuals being confined due to the pandemic. Despite the extraordinary national measures in combating the outbreak, the success or failure of these efforts is largely dependent on public behavior. Public adherence to preventive measures established by the government is of prime importance to prevent the spread of the disease. Adherence is likely to be influenced by the public's knowledge and attitude toward COVID-19. Evidence shows that public knowledge is important in tackling pandemics (44). Hence, it is essential to have COVID-19 related knowledge, the populations attitudes, and their practices as a part of the surveillance system. By assessing public awareness and knowledge about the coronavirus, deeper insights into existing public perception and practices can be gained, thereby helping to identify attributes that influence the public in adopting healthy practices and responsible behaviors (44). Assessing public knowledge is also important in identifying gaps and strengthening ongoing prevention efforts. Combining multifaceted subjective and objective data using the DIK (Data, Information, Knowledge) framework will help generate the risk profile of an individual and the community. A human-centered approach combined with information processing theory and humanistic, behavioral learning, and self-efficacy theories will facilitate feedback based on user engagement, task analysis and requirements, and on individual classification into prevention, monitoring, and referral ad management categories. These would be evidence based and would guide the development of programs, policies, and interventions driven by data (Figure 8). In addition, population health informatics enabled surveillance systems should guide outcome assessment of variables including process, clinical, quality of life, cost effectiveness, and longevity (Figures 8, 9).

## Discussion—Public Health and Policy Implications

At present, India faces a triple burden of diseases—infectious diseases, the challenge of non-communicable diseases, and emergence of the COVID-19 pandemic. Topping the disease caseload, challenges unique to India in these trying times discussed in this paper make tackling the pandemic complex, unless the existing health infrastructure in India that is already over-stretched is strengthened to face these challenges in the 21st century.

Prior to commencement of lockdown four in India on May 18, 2020, the country was focusing on national decisions; it then switched to a state-based approach where each state devised its own policies and regulations. States implemented zone-based policies depending on the extent of cases. Bearing in mind this pandemic, extreme vulnerabilities were exposed in a host of aspects surrounding the coping mechanisms of a country, be it the drug supply chain of a nation, information systems, or government compositions. These interdisciplinary shortcomings could possibly present similar, if not worse, implications in a future health disaster; and as public health professionals, our responsibility to safeguard the vulnerable populations in that future are looming large. Key findings in literature framing the problem of structural determinants of health disparities in a pandemic focus on reliability of data, national policy, and surveillance characteristics among social inequality, interpersonal relationships, and biopsychosocial-related weathering. The conceptual model (Figures 8, 9) includes elements of an integrated cumulative pathways approach, which posits that efforts to support these pathways will result in a strapping coping system for future health disasters. A shortage of PPE in India and fragility of the medical equipment stockpile was a result of the accumulated lack of effort over time, indicating that little attention is paid to factors that undermine efforts to protect frontline health-care workers. The addition of public health advisors in the Indian cabinet will enhance administrative response efforts surrounding stocks of health supplies and ensure that front-line workers have all the essential equipment to protect themselves during this pandemic.

Research studies should focus on learnings from the ongoing challenges and the knowledge generated from this research can be utilized to strengthen the information economy and provide evidence-based solutions. As discussed earlier in this paper, we made suggestions about the stable increase in the recovery rate of Indians for COVID-19, possibly hinting at accrediting a higher recovery rate due to natural preventive medicine practices than that of its counterparts. Studies should focus on these ancient practices of the Indian heritage that assist infected patients in fighting the effects of this disease. Efforts to create integrated systems for data management are also calling for action as lack of testing data during the current pandemic has caused misreporting and underestimation of the severity of this disaster. Access to testing data by the state in real time, hospitalizations, availability of beds per state, and supplies available to each state should be made available to the public. Increasing usage of mobile phones can facilitate digital interventions to gather and deploy uniform information to ~1.3 billion citizens. Using digital platforms will subjugate data management and health information systems in a close knit manner. By using datasets from around the world, this paper analyzed how the total number of COVID-19 cases and the number of active, recovered, and deceased cases grew, and inferences were made on the impact of the pandemic in India. Daily spikes are credited to human psychology along with outcomes of the lockdown in varying sociocultural settings. From this study, we can suggest that governments of densely populated developing countries such as India implement changes to policies and lockdown guidelines based on the nature of the outbreak in various regions and focus on preparedness. It is important to formulate tools for better district-level planning and prioritization as well as effective allocation of resources (40). Foreseen challenges related to the surveillance system proposed here mainly include the availability of publicly available COVID-19 data in a timely manner, the reporting of cases and deaths related to COVID-19 usually done in an routinely manner, and the lack of availability of advisory and policy data in response to COVID-19 in a homogeneous manner. These limitations stand for countries like Vietnam, Pakistan, and India and might possibly obstruct surveillance.

Additionally, the “information economy” has contributed to confusion and panic surrounding COVID-19, with instances of unreliable news reports, glitches in filtering, flagging of information that is valid, and world leaders diminishing the coronavirus crisis. Recent research of existing websites disseminating information about the pandemic indicates that current studies are important as they show critical gaps in the information about COVID-19. But it warns that “there is a lack of good quality websites with useful and quality novel coronavirus-related health information” (45). The coronavirus pandemic is a new social phenomenon too, which demands a need for better communication of clear authoritative information. According to Alang (2020), “The nuanced differences between social distancing, self-isolation and quarantining, for example, all need to be communicated to and learned by society at large” (45). Unfortunately, many popular media channels in India seem to go against this grain. Citizens in India require accurate information, with cross-section collaborations used to monitor and filter false information (46) during a public health emergency.

## Limitations and Conclusion

We understand that correlation does not necessarily translate to causation. However, we are able to offer concrete theories of possible associations between variables—protests impacting the incidence of COVID-19 and period prevalence rates or laboratory testing numbers indicating higher incidence. Additionally, we also have reservations about publicly available testing data in India. Expensive or unreliable tests, slow turnaround time, and poor efficiency of testing raises questions about the veracity of public data. Information on real-time state-wise testing, hospitalizations, state-wise availability of beds, and supplies available to each state were also not accessible. This commentary also acknowledges the slow spread of this epidemic in India but provides little insight into this epidemiologic curiosity, however, a deeper understanding of this unnatural trend is a possible investigation.

The first pandemic declared by the WHO in the 21st century has offered us a singular learning opportunity. We are at a unique crossroads with a new world of social media and quickly evolving generations. It is important to understand how this sort of population responds to stimuli—in this case a global pandemic caused by a highly contagious virus. Current literature has not yet addressed how social media affects the pandemic in India. Is it a boon or a bane? It would also be interesting to study the links between India's young median age and the country's recovery rate. These research opportunities will help us build greater preparedness for any such future health impacts. At this moment, ongoing reforms toward structural economic policy in India and other such settings must continue. A robust urban employment scheme to support the vulnerable populations is seen as an essential next effort. Microeconomic policies to ameliorate the distress caused by the pandemic along with human rights should guide the COVID-19 responses of the devolution. Equality and non-discrimination are principles to create rightful support opportuities for vulnerable groups.

## Author Contributions

AJ conceptualized the research paper and contributed to manuscript writing. AM contributed to drafting the paper, manuscript writing, data analysis and interpretation, and critical editing. SA contributed to data gathering. AG contributed to manuscript writing and providing feedback. All authors contributed to the article and approved the submitted version.

## Conflict of Interest

The authors declare that the research was conducted in the absence of any commercial or financial relationships that could be construed as a potential conflict of interest.
